# Deformation Performance of Longitudinal Non-Uniformly Corroded Reinforced Concrete Columns

**DOI:** 10.3390/ma17215303

**Published:** 2024-10-31

**Authors:** Guoyao Sun, Huanjun Jiang

**Affiliations:** 1 State Key Laboratory of Disaster Reduction in Civil Engineering, Tongji University, Shanghai 200092, China; 2 Department of Disaster Mitigation for Structures, College of Civil Engineering, Tongji University, Shanghai 200092, China

**Keywords:** corroded RC column, non-uniform corrosion, FEM, deformation performance

## Abstract

Due to the complexity of the marine corrosive environment, the rebar corrosion in reinforced concrete (RC) bridge piers is usually longitudinal non-uniform. However, the study on the mechanical behavior of longitudinal non-uniformly corroded RC structural members is very limited. To systematically study the deformation performance of the longitudinal non-uniformly corroded RC columns, the finite element models of 106 RC columns with different parameters were established using the commercial software ABAQUS 2016. The effects of the height of the bottom section (represented in the text by the variable “position”), the length, and the rebar corrosion ratio of the corroded segment on the deformation performance of the longitudinal non-uniformly corroded RC columns were analyzed. It is found that the change in the position of the corroded segment on the column may change the most unfavorable section of the column and the failure mode. The length of the corroded segment significantly affects the yield deformation. The ultimate plastic deformation increases with the increase of position or length of the corroded segment. With the increase of rebar corrosion ratio of the corroded segment, the ultimate plastic deformation decreases.

## 1. Introduction

The durability damage to reinforced concrete (RC) structures, primarily due to material aging, influence of the external environment, improper use, etc., is an important cause of structural performance degradation, and rebar corrosion is recognized as the chief factor causing insufficient durability [1]. According to the previous research results, rebar corrosion can result in the material properties degradation of RC structures, including the loss of rebar area, cracking, delamination, and spalling of concrete cover, and the strength deterioration of the bond between the rebar and the concrete. Regarding RC columns’ mechanical performance, rebar corrosion can result in changes in the failure mode and reductions in bearing capacity and ductility [2,3,4,5].

Previous research mainly focuses on the mechanical behavior of uniformly corroded RC columns. However, rebar corrosion in the naturally corrosive environment is mostly non-uniform due to the long service time and the complex environment. Due to the ocean’s tidal phenomenon, bridge piers in coastal areas are in several different corrosive environments, such as the marine atmosphere zone, splash zone, tide zone, and immersion zone [6]. The complexity of the natural corrosive environment results in longitudinal non-uniform corrosion of the rebar in the bridge piers. Zhou et al. [7] investigated the failure of an RC bridge in eastern Shenzhen due to rebar corrosion in the marine environment. Measurements of the rebar corrosion ratio indicated that there was a non-uniformity of corrosion along the longitudinal direction of the rebar as the vertical corrosion environment varied.

The study on the mechanical behavior of longitudinal non-uniformly corroded RC columns is very limited. Considering the influence of splash zone and tide zone on the longitudinal non-uniform corrosion of the rebar of the bridge pier, Guo et al. [8], Yuan et al. [9], and Zhou et al. [10] conducted cyclic loading tests on longitudinal non-uniformly corroded RC columns with different levels of corrosion. These studies demonstrated that the peak load, ductility, and energy dissipation capacity of the columns decreased significantly with the increase in rebar corrosion ratio, and the location of the plastic hinge shifted from the bottom of the column to the corrosion segment. However, previous studies focused on the effect of different rebar corrosion ratios on non-uniformly corroded RC columns with one fixed position and length of the corroded segment, and no study has been carried out to investigate the effect of different position and length of the corroded segment. Actually, the position and length of the corroded segment vary due to the variation of the vertical corrosion environment. Therefore, it is necessary to systematically investigate the effects of the position and length of the corroded segment on the mechanical behavior of the corroded RC columns.

In this study, numerical analysis was carried out with the aid of the commercial software ABAQUS to investigate the effects of the position, the length, and the rebar corrosion ratio of the longitudinal non-uniformly corroded segment on the deformation performance of the corroded RC columns.

## 2. Materials and Methods

### 2.1. Numerical Model

The numerical modeling of longitudinal non-uniformly corroded RC columns was conducted using the finite element analysis software ABAQUS 2016. ABAQUS is a large and general-purpose finite element software for engineering simulation, which is widely used for numerical simulation in civil engineering. In comparison with other numerical software, ABAQUS is capable of refined finite element modeling and has the material types and element types suitable for the simulation of concrete, rebar, and bond between concrete and rebar for RC structural members, which is necessary for the modeling of longitudinal non-uniformly corroded RC columns. In this study, the three-dimensional FE model was adopted.

The separate modeling method was applied in the numerical simulation of the mechanical behavior of corroded RC columns in this study, by which the rebar and concrete are modeled as independent instances, and the independent instances of rebar and concrete were assigned corresponding material properties and element type, and the bond between rebar and concrete was simulated using a connector in this study.

The concrete was modeled by adopting the 8-node 3-D solid element (C3D8R). The concrete tensile and compressive constitutive relationship recommended by the Chinese Code for Design of Concrete Structures [11] was adopted. Corrosion products of the rebar can cause the cracking of the concrete cover, resulting in the strength loss of the concrete cover. Vecchio et al. [12] treated cracked concrete as a new material in their study and proposed constitutive relationships for cracked concrete based on the modified compression-field theory and material mechanical test. Combining the model proposed by Vecchio et al. and the studies for concrete cracking caused by rebar corrosion, Coronelli and Gambarova [13] proposed the reduced strength model of concrete cover suffering corrosion damage, and the experimental results of corroded RC beams verified the model. This model was applied in the numerical simulation of corroded RC columns [14,15,16,17]. Therefore, the reduced strength model of concrete cover suffering corrosion damage proposed by Coronelli and Gambarova [13] was adopted in this study as follows:

(1) $$f_{c}^{*}=\frac{f_{c}}{1+K\varepsilon_{1}/\varepsilon_{c0}}$$

where *f_c_* is the compressive strength of concrete; f_c_^*^ is the reduced compressive strength of concrete after corrosion damage; *K* is the coefficient related to bar roughness and diameter, and for medium-diameter ribbed bars, it is taken as 0.1, as suggested by Cape [18]; *ε_c_*_0_ is the strain at the peak compressive stress; and *ε*_1_ is the average (smeared) tensile strain in the cracked concrete at right angles to the direction of the applied compression.

The rebar was modeled by the 2-node linear beam element (B31). The model that can describe the mechanical properties of naturally corroded rebar, proposed by Zhang et al. [19], was adopted in this study as follows:

(2) $$A_{c}=\left(1-\eta \right)A_{0}$$

(3) $$f_{yc}=\frac{\left(1-1.049\eta \right)}{1-\eta }f_{y0}$$

(4) $$\varepsilon_{suc}=e^{-2.501\eta }\varepsilon_{su0}$$

where *A*_0_ is the original cross-sectional area of uncorroded rebar; *A_c_* is the reduced cross-sectional area of corroded rebar; *f_y_*_0_ is the original yield strength of uncorroded rebar; *f_yc_* is the reduced yield strength of corroded rebar; *ε_su_*_0_ is the original ultimate strain of uncorroded rebar; *ε_suc_* is the reduced ultimate strain of corroded rebar; and *η* is the rebar corrosion ratio defined as the ratio of the mass loss of corroded rebar to the original mass of the uncorroded rebar.

The bond between the rebar and the concrete was modeled using a connector type CARTESIAN, which provides a connection between two nodes without kinematic constraints. The expression of the bond stress-slip relationship between the corroded rebar and the concrete is:

(5) $$\tau \left(s\right)=\beta \tau_{0}\left(s\right)$$

where *τ*_0_(*s*) is the bond stress-slip relationship between the uncorroded rebar and the concrete, and the bond stress-slip relationship recommended by the Chinese Code for Design of Concrete Structures [11] was adopted; *β* is the normalized bond strength coefficient defined as the ratio of the bond strength at a certain corrosion ratio *η* to the bond strength of uncorroded rebar, and the expression to determine *β* which is proposed by Bhargava et al. [20] based on large quantities of experiment data was adopted as follows:

(6) $$\beta =\left\{\begin{array}{cc} 1, & \eta \leq 1.5\%\\ 1.192e^{-11.7\eta }, & \;\;\eta >1.5\% \end{array}\right..$$

**Figure 1:**
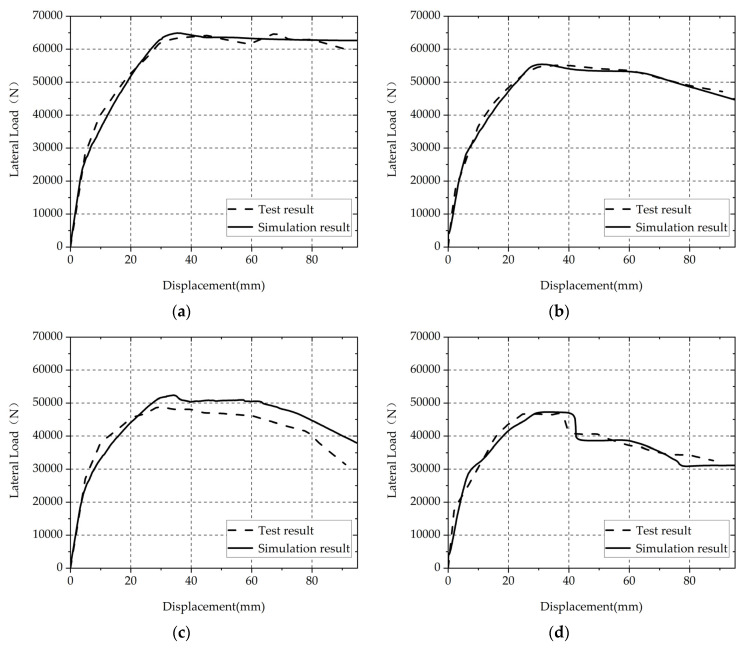
Comparison of load-displacement relationship curves between the test data and the numerical simulation results: (**a**) S1; (**b**) S2; (**c**) S3; (**d**) S4.

To validate the accuracy of the three-dimensional FE model with the adopted corrosion damage models, numerical models were established in ABAQUS to simulate the mechanical performance of longitudinal non-uniformly corroded RC columns tested by Guo et al. [8]. Figure 1 shows the comparison of load-displacement relationship curves between the test data and the numerical simulation results. The comparison between test and simulation results is shown in Table 1. As shown in Figure 1 and Table 1, the simulation results are in good agreement with the test results in comparing the load-displacement relationship curves and the error of peak load and ultimate deformation.

### 2.2. Columns Designed for Analysis

In order to systematically investigate the deformation performance of longitudinal non-uniformly corroded RC columns, the main parameters which may affect the mechanical properties of RC columns were determined at the first stage.

The design data from a large number of bridge pier studies was collected, and the key design parameters that mainly affect the bearing capacity, deformation performance and failure mode of the piers were extracted, including shear-to-span ratio, axial compressive ratio, longitudinal reinforcement ratio, and volumetric stirrup ratio. Based on the data summary for the key design parameters, the representative parameters for each key design parameter were adopted. According to the relevant specifications, representative columns corresponding to combinations of representative parameters for different key design parameters were designed.

Combining the provisions of relevant specifications for the corrosive environment and the design water level, the measured values of tidal difference data and wave height data at tide gauge stations along the coast of China from a large number of studies were collected, and the key corrosion parameters that mainly affect the mechanical behavior of the piers were extracted, including the position and the length of corroded segment corresponding to various marine corrosive environments. Based on the measured corrosion ratio data of the previous studies on the rebar corrosion of RC structures in marine corrosive environments, the rebar corrosion ratio is considered a key corrosion parameter, and the main range of rebar corrosion ratio is obtained. Based on the data summary for the key corrosion parameters, representative parameters for each key corrosion parameter were adopted. The combinations of representative parameters for different key corrosion parameters are used as the corrosion scenarios for the study of the deformation performance of longitudinal non-uniformly corroded RC columns.

In this study, columns with the same combination of representative key design parameters and all corresponding corrosion scenarios were adopted to investigate the effects of longitudinal non-uniform corrosion parameters on the deformation performance of the columns. Since the full height of this set of columns is in the potential vertical range of the splash and tide zone, the lengths of the corroded segment in this study were taken as specific ratios of the column height for investigation of mechanical behavior instead of the representative parameters based on data summary.

According to the current Chinese Code for Seismic Design of Buildings [21] and the Chinese Specifications for Seismic Design of Highway Bridges [22], 106 longitudinal non-uniformly corroded RC columns were designed and analyzed. The variable parameters included the position *p*, the length *L*, and the rebar corrosion ratio *η* of the longitudinal non-uniformly corroded segment of the columns. According to the previous research [7,9], the segment of bridge piers in the marine atmosphere zone and the immersion zone were considered to have little rebar corrosion, simplified to be uncorroded in the study, and the segment of bridge piers in the splash zone and the tide zone were considered to have serious rebar corrosion, simplified to be corroded in the study.

**Figure 2:**
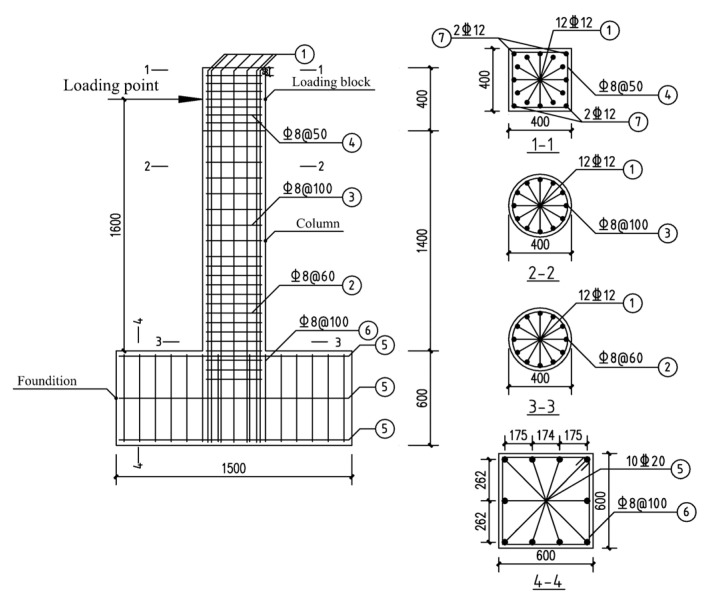
The geometric dimensions and rebar details of the columns (unit: mm).

**Figure 3:**
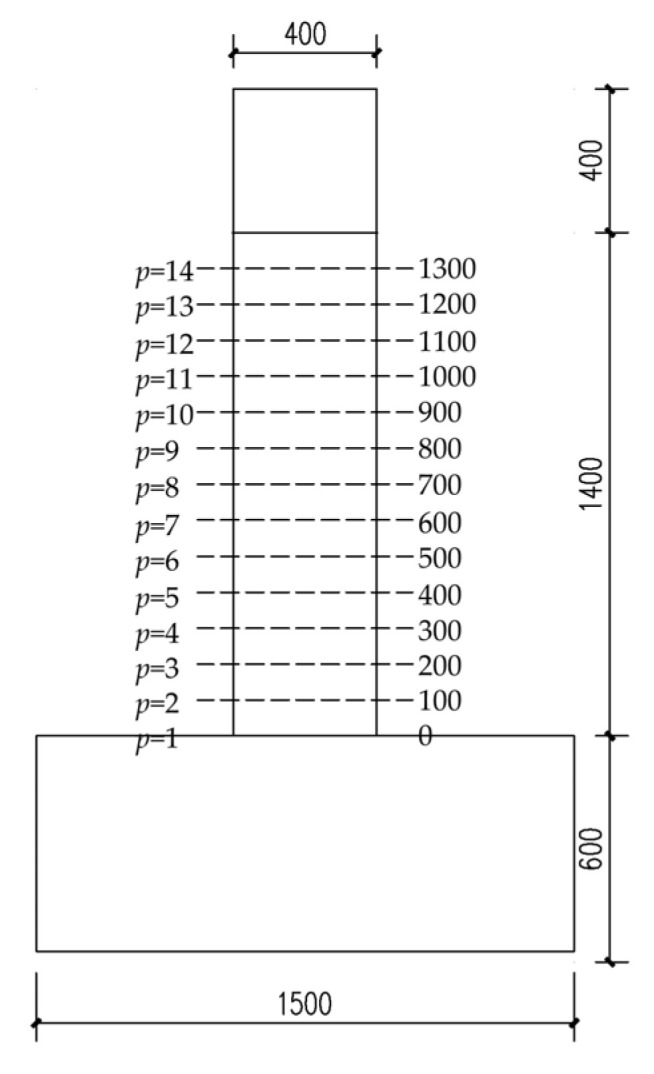
The position of the corroded segment (unit: mm).

**Figure 4:**
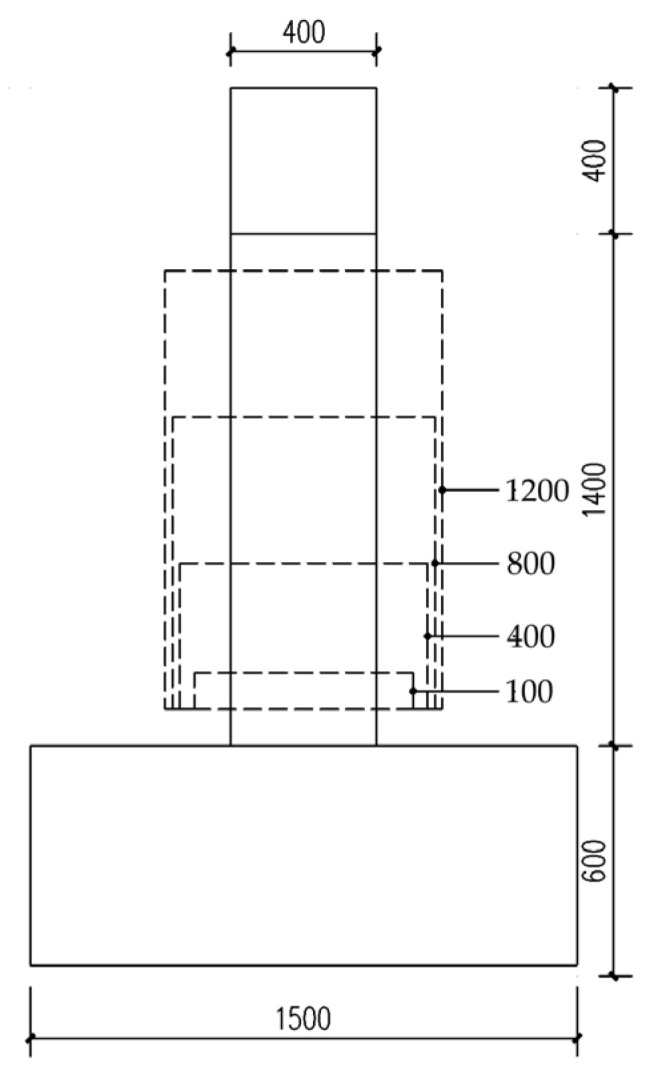
The length of the corroded segment (unit: mm).

The geometric dimensions and rebar details of the longitudinal non-uniformly corroded columns are shown in Figure 2. The yield strength of the longitudinal rebar and the stirrup were 400 MPa and 335 MPa, respectively. The compressive strength of the concrete was 20.1 MPa. As the axial compressive ratios of the bridge piers are small, the axial compressive ratio of the columns in this study was 0.2. According to the degree of corrosion of rebar in real structures, the corrosion ratio of longitudinal rebar in corroded segments ranged from 0% to 30% at an interval of 10%. According to the measurements, the stirrup corrosion ratio was taken as 2.5% greater than the corrosion ratio of the longitudinal rebar in the corresponding corrosion level [7]. Because the corroded segment has a certain length in the longitudinal direction of the column and the position parameter of the corroded segment only needs the position of one section in the longitudinal direction as the representative, the position of the bottom section of the corroded segment is considered as the position of the corroded segment. The positions of the corroded segment were taken as 1 to 14, and the positions increased upward in 100 mm increments measured from the top surface of the foundation, as shown in Figure 3. As shown in Figure 4, the length of the corroded segment was taken as 1/16, 1/4, 1/2, and 3/4 times the distance from the loading point to the top surface of the foundation, i.e., 100 mm, 400 mm, 800 mm, and 1200 mm, respectively. According to the length relationship, *p* and *L* should meet the following requirements:

(7) $$100\left(p-1\right)+L\leq 1400$$

The values considered for the position, the length, and the rebar corrosion ratio of the corroded segment are shown in Table 2.

The columns were numbered as C-*p*-*L*-*η*. For example, C-4-400-20 represents the column with *p* of 4, *L* of 400 mm, and *η* of 20%. Additionally, an uncorroded column numbered as C-0-0-0 was designed as a reference model.

## 3. Results and Discussion

**Figure 5:**
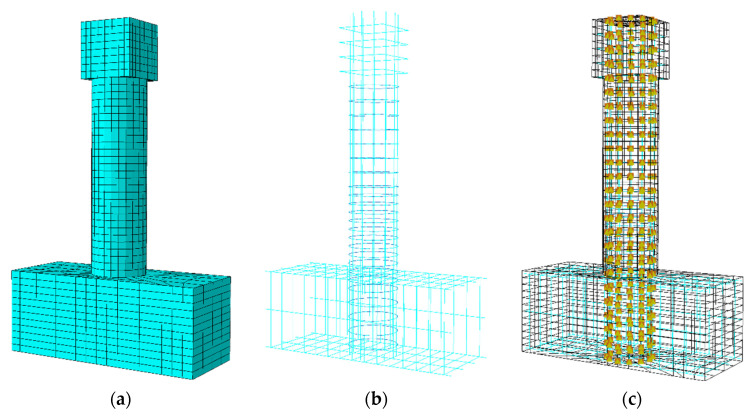
FE model of the columns. (**a**) Concrete; (**b**) rebar; (**c**) connector.

The FE models for the columns designed above were established by adopting the verified numerical modeling method, as shown in Figure 5. The FE model was fixed at the base. The constant vertical load and the horizontal displacement were applied at the loading top block. The FE analysis results of each column, such as the load-displacement relationship curve and stress/strain cloud maps of concrete and rebar, were obtained. The column is considered to reach its ultimate limit state when any of the following three conditions occurs: 15% reduction in load-carrying capacity of the column, fracture of tensile longitudinal rebar of the column, and crushing of core concrete of the column. At the ultimate limit state, the horizontal displacement at the loading point of the column is defined as the ultimate deformation. The ultimate deformation was divided into two parts: yield deformation and ultimate plastic deformation for the investigation. When the outermost tensile longitudinal rebar of the column reaches yield, the horizontal displacement at the loading point of the column is defined as the yield deformation. The deformation difference between the yield deformation and the ultimate deformation is defined as the ultimate plastic deformation. Based on the FE simulation results, the influence of the position, the length, and the rebar corrosion ratio of the corroded segment on the deformation performance of the columns were analyzed, respectively.

### 3.1. Influence of the Position of Longitudinal Non-Uniformly Corroded Segment

**Figure 6:**
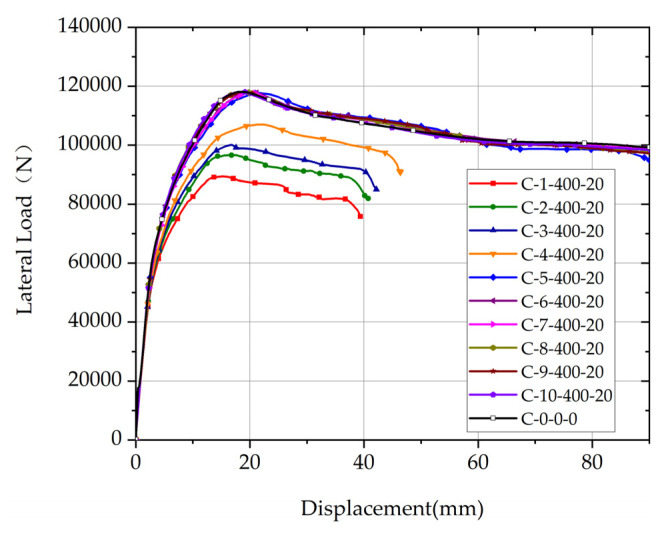
Comparison of load-displacement relationship curves of columns with different positions of the corroded segment and the uncorroded column.

Comparison of load-displacement relationship curves of columns with different positions of the corroded segment and the uncorroded column are shown in Figure 6. The length of the corroded segment is 400 mm, the rebar corrosion ratio of the corroded segment is 20%, and the position of the corroded segment of each column varies from 1 to 10.

As shown in Figure 6, the corroded segment’s position significantly affects the load-displacement relationship curve of the columns. The load-displacement relationship curves of the non-uniformly corroded columns with the position of the corroded segment *p* exceeding *p_c_*, which indicates a critical position, are almost identical to that of the uncorroded column C-0-0-0. On the other hand, the load-displacement relationship curves of the non-uniformly corroded columns, with *p* not exceeding *p_c_*, are significantly different from those of the uncorroded column C-0-0-0. Compared with C-0-0-0, the deformation capacity of non-uniformly corroded columns is lower. The ultimate deformation of non-uniformly corroded columns increases significantly with the increase of *p*. Therefore, *p_c_* is named as the corroded critical position. While the length and the rebar corrosion ratio of the corroded segment are taken as different values, respectively, the effect of the position of the corroded segment on the load-displacement relationship curve is consistent with the conclusion of Figure 6.

The reason for the significant effect of the position of the corroded segment on the load-displacement relationship curve of the column is that the yield condition of the rebar at the bottom section of the corroded segment determines the most unfavorable section of the column. With *p* not exceeding *p_c_*, the bending moment at the bottom section of the corroded segment is so large that the rebar can yield at this section, and, therefore, this section is the most unfavorable section of the column. With *p* exceeding *p_c_*, the bending moment at the bottom section of the corroded segment is not so large that the rebar cannot yield at this section, and the rebar at the bottom of the column can yield normally, therefore the section at the bottom of the column is the most unfavorable section of the column.

It can be found that the ultimate deformations of columns with *p* not exceeding *p_c_* show a significant reduction compared to that of the uncorroded column, and the ultimate deformations of columns with *p* exceeding *p_c_* are almost identical to that of the uncorroded column. Therefore, in the following section, the parametric analysis of the ultimate deformations of non-uniformly corroded columns focuses on columns with *p* not exceeding *p_c_*.

**Figure 7:**
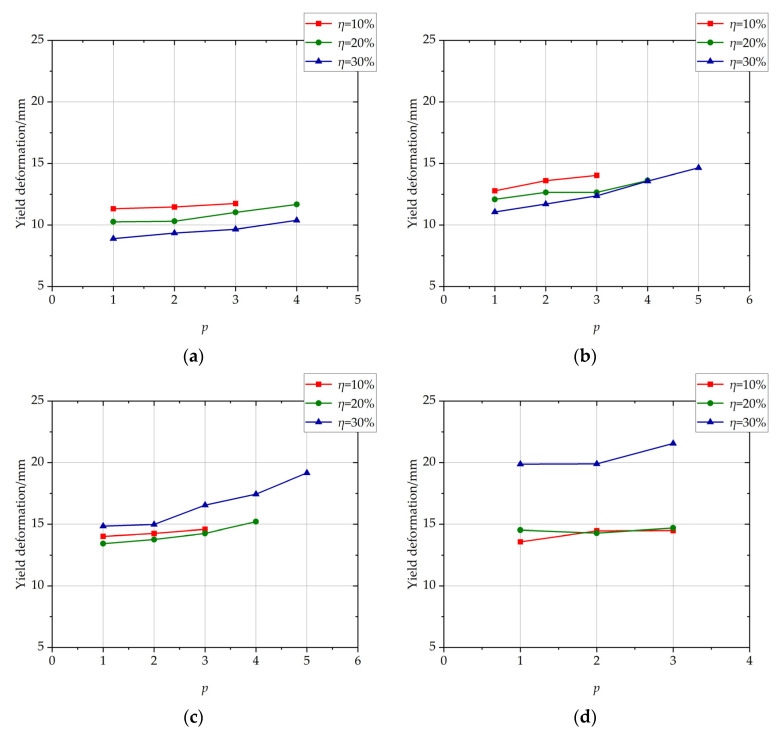
The influence of the position of the non-uniformly corroded segment on yield deformation of columns with different *η* values. (**a**) *L* = 100 mm; (**b**) *L* = 400 mm; (**c**) *L* = 800 mm; (**d**) *L* = 1200 mm.

The influence of the position of non-uniformly corroded segment on yield deformation of columns with different *η* values is shown in Figure 7. With the increase in the position of the corroded segment, the yield deformation increases slightly and approximately linearly. From Figure 7, it is found that with the increase of the length of the corroded segment, the blue curve, which represents the curve with *η* = 30%, gradually rises from being lower than the other two curves to be higher than those caused by the accumulation of bond deformation with the high corrosion ratio along the length of the corroded segment, as discussed in detail in Section 3.2.

**Figure 8:**
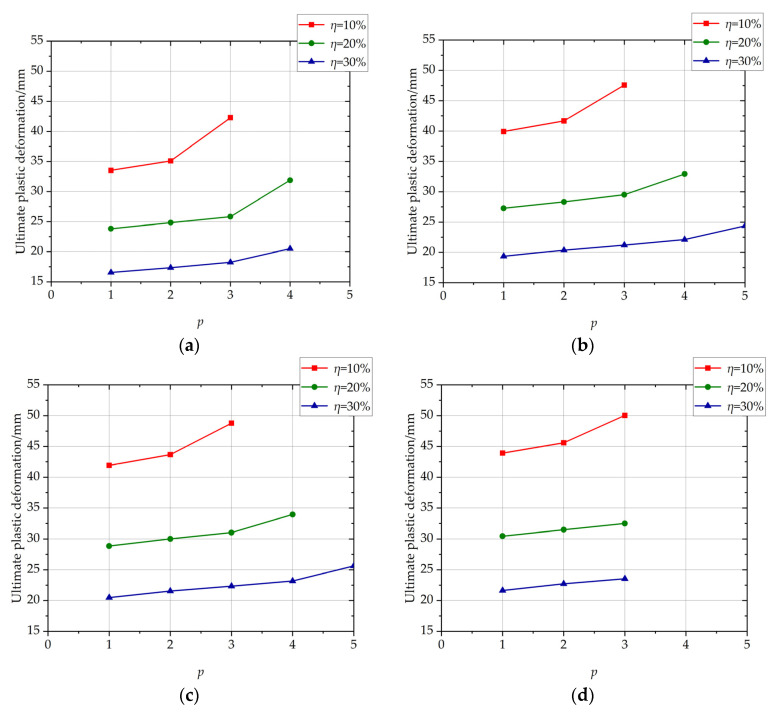
The influence of the position of the non-uniformly corroded segment on ultimate plastic deformation of columns with different *η* values. (**a**) *L* = 100 mm; (**b**) *L* = 400 mm; (**c**) *L* = 800 mm; (**d**) *L* = 1200 mm.

**Figure 9:**
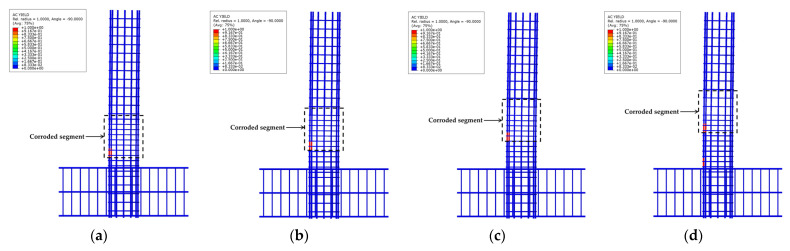
Cloud map of the rebar-yielding of the columns with different positions of the corroded segment at peak load. (**a**) C-2-400-30; (**b**) C-3-400-30; (**c**) C-4-400-30; (**d**) C-5-400-30.

The influence of the position of the non-uniformly corroded segment on the ultimate plastic deformation of columns with different *η* values is shown in Figure 8. With the increase of position of the corroded segment from 1 to *p_c_* − 1, the ultimate plastic deformation increases approximately linearly. With the increase of position of the corroded segment from *p_c_* − 1 to *p_c_*, the ultimate plastic deformation increases with a large increment. The cloud map of rebar-yielding of the columns with different positions of corroded segments at peak load is shown in Figure 9. With the position of the corroded segment from 2 to 4, which is less than *p_c_*, the outermost tensile longitudinal rebar at the bottom section of the corroded segment enters plasticity. With *p* of 5, which is *p_c_* in this condition, the outermost tensile longitudinal rebar at the bottom section of the corroded segment and the column enter plasticity. The reason for the large increment is that the outermost tensile longitudinal rebar at the bottom section of the column enters plasticity with *p* = *p_c_* at the ultimate limit state, and this plastic deformation at the bottom segment of the column is an extra part relative to the columns with *p* < *p_c_* whose rebar at the bottom section of the column does not yield.

### 3.2. Influence of the Length of Longitudinal Non-Uniformly Corroded Segment

**Figure 10:**
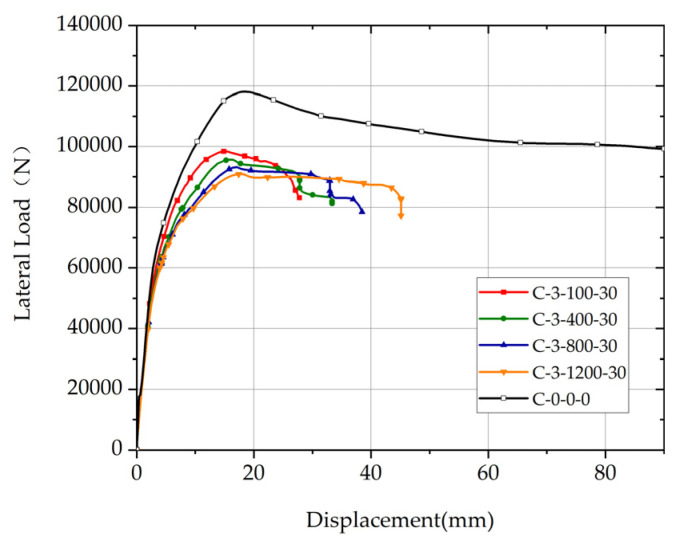
Comparison of the load-displacement relationship curves of columns with different lengths of the corroded segment and the uncorroded column.

Comparison of load-displacement relationship curves of columns with different lengths of the corroded segment and the uncorroded column are shown in Figure 10. The position of the corroded segment is 3, the rebar corrosion ratio of the corroded segment is 30%, and the lengths of the corroded segment of columns are 100 mm, 400 mm, 800 mm, and 1200 mm. The ultimate deformation of non-uniformly corroded columns increases significantly with the increase of *L*.

**Figure 11:**
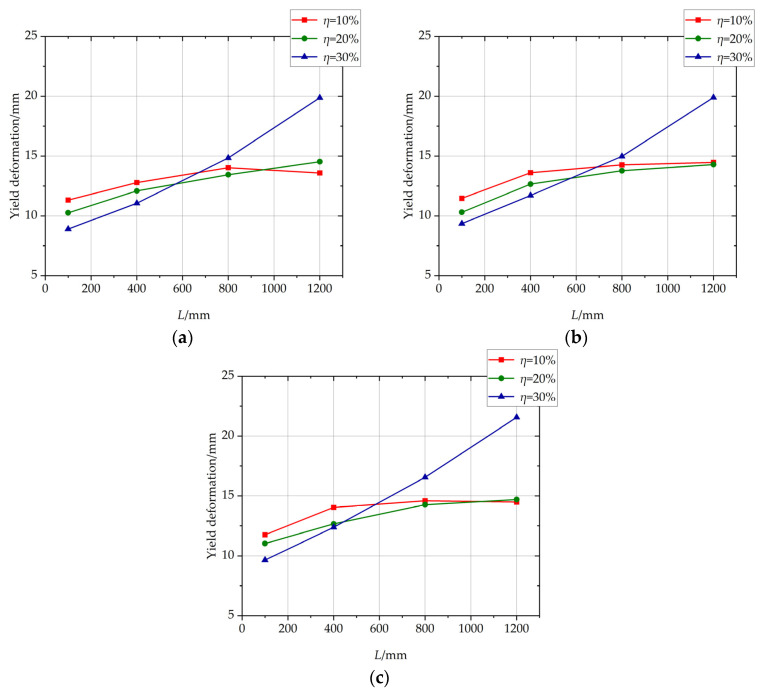
The influence of the length of non-uniformly corroded segment on yield deformation of columns with different *η* values. (**a**) *p* = 1; (**b**) *p* = 2; (**c**) *p* = 3.

The influence of the length of non-uniformly corroded segment on the yield deformation of columns with different *η* values is shown in Figure 11. For columns with *η* of 10% or 20%, with the increase in the length of the corroded segment, the yield deformation increases, and the ascending trend of the yield deformation slows down. For columns with *η* of 30%, with the increase in the length of the corroded segment, the yield deformation increases approximately linearly.

**Figure 12:**
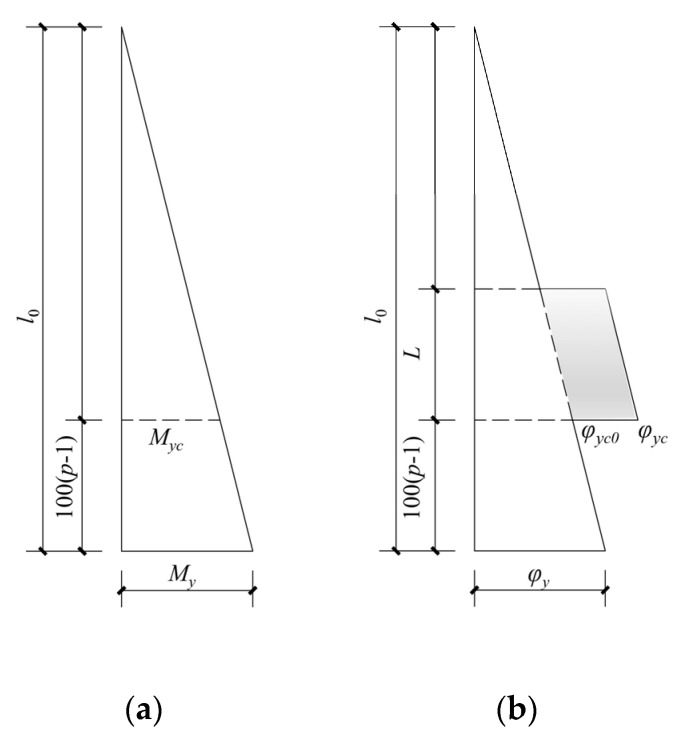
The distribution of the sectional bending moment and sectional curvature along the longitudinal direction of the non-uniformly corroded columns at the rebar-yielding state. (**a**) Sectional bending moment; (**b**) sectional curvature.

The distribution of sectional bending moment along the longitudinal direction of the non-uniformly corroded columns at the rebar-yielding state is shown in Figure 12a. The distribution of sectional curvature along the longitudinal direction of the non-uniformly corroded columns at the rebar-yielding state is shown in Figure 12b. The additional curvature, which is the shaded part shown in Figure 12b, is produced by the material degradation of RC columns due to rebar corrosion. The curvature is length-integrated along the longitudinal direction of the column, and the following equation determines the corresponding theoretical yield deformation Δ_y_:

(8) $$\begin{array}{cc} \Delta_{y} & =\frac{\varphi_{y}l_{0}^{2}}{3}+\left(\varphi_{yc}-\varphi_{yc0}\right)L\left\{\left[l_{0}-100\left(p-1\right)\right]-0.5L\right\}\\ & =\frac{\varphi_{y}l_{0}^{2}}{3}-\left(\varphi_{yc}-\varphi_{yc0}\right){\left\{L-\left[l_{0}-100\left(p-1\right)\right]\right\}}^{2}+\left(\varphi_{yc}-\varphi_{yc0}\right){\left[l_{0}-100\left(p-1\right)\right]}^{2} \end{array}$$

From Equation (8), the relationship between *L* and Δ*_y_* is described by a quadratic function, Δ*_y_* increases, and the ascending trend slows down with the increase of the length of the corroded segment, which is consistent with the parametric investigation results by the FE simulation with the corrosion ratio of 10% or 20% in Figure 11. The reason for this phenomenon is that the quadratic function relationship with *L* determines the deformation produced by additional curvature in the corroded segment because it is calculated by additional curvature length integrated within *L* along the longitudinal direction of the column. The main reason for the difference between yield deformation versus length of corroded segment relationship with *η* = 30% and that with *η* = 10% or 20%, as shown in Figure 10, is that the bond between concrete and rebar in corroded segment degrades seriously at high rebar corrosion ratio level, and bond–slip deformation increases so much that it becomes an important part of yield deformation. With the increase in the length of the corroded segment, the length of the bond degradation increases, and the bond-slip deformation of columns at yield state increases significantly, which affects yield deformation versus length of corroded segment relationship with *η* = 30%.

**Figure 13:**
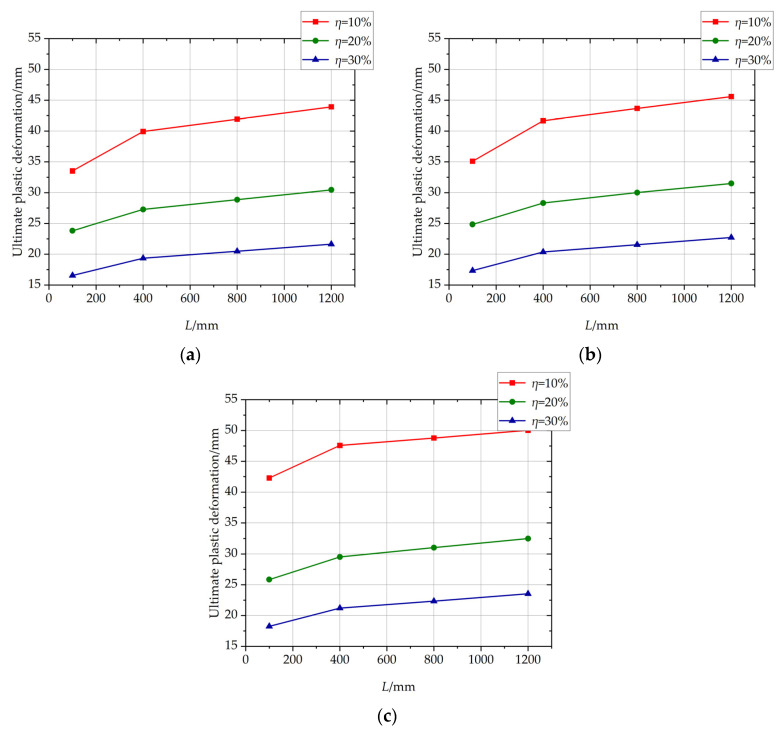
The influence of the length of the non-uniformly corroded segment on the ultimate plastic deformation of the columns with different *η* values. (**a**) *p* = 1; (**b**) *p* = 2; (**c**) *p* = 3.

**Figure 14:**
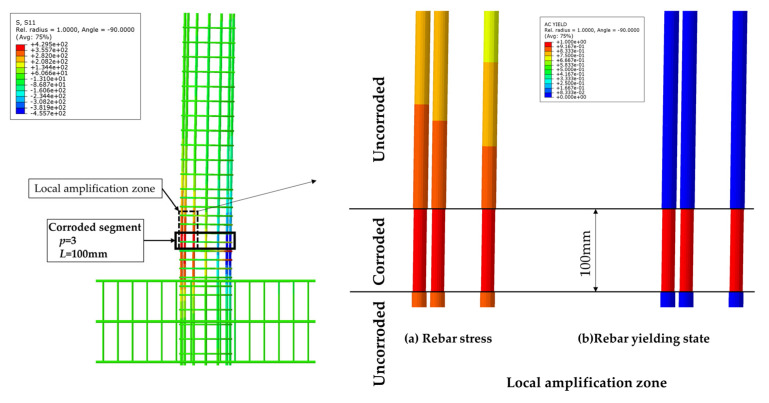
The cloud map of the rebar stress of C-3-100-20 and the cloud map of the rebar stress and the rebar-yielding of the local amplification zone.

**Figure 15:**
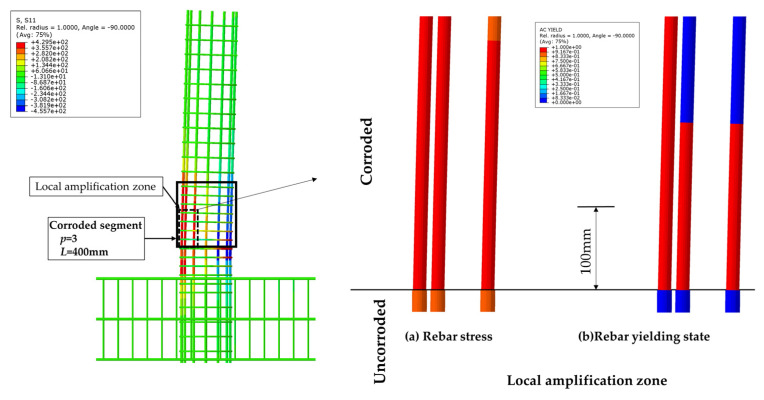
The cloud map of the rebar stress of C-3-400-20 and the cloud map of the rebar stress and the rebar-yielding of the local amplification zone.

The influence of the length of the non-uniformly corroded segment on the ultimate plastic deformation of columns with different *η* values is shown in Figure 13. With the increase of the length of the corroded segment from 100 mm to 400 mm, the ultimate plastic deformation increases with a large increment. With the increase of the length of the corroded segment from 400 mm to 1200 mm, the ultimate plastic deformation increases slightly and approximately linearly. The large increment is because the length of the corroded segment affects the development of the plastic hinge. The cloud map of the rebar stress of C-3-100-20 and the cloud map of rebar stress and the rebar-yielding of the local amplification zone, is shown in Figure 14. The cloud map of the rebar stress of C-3-400-20 and the cloud map of the rebar stress and the rebar-yielding of the local amplification zone is shown in Figure 15. As the length of the corroded segment is small, the outermost tensile longitudinal rebar in the corroded segment enters plasticity for the whole length of the corroded segment, and the plastic hinge is formed for the whole length of the corroded segment, as shown in Figure 14. The rebar outside the corroded segment is uncorroded, which has higher mechanical performance than the corroded rebar and does not enter plasticity. Constrained by the length of the corroded segment, the plastic hinge is not fully developed, and the corresponding ultimate plastic deformation is small with a small plastic hinge length. As the length of the corroded segment is large, the plastic hinge fully develops, and the ultimate plastic deformation is large with the fully developed plastic hinge length, as shown in Figure 15.

### 3.3. Influence of the Rebar Corrosion Ratio of Longitudinal Non-Uniformly Corroded Segment

**Figure 16:**
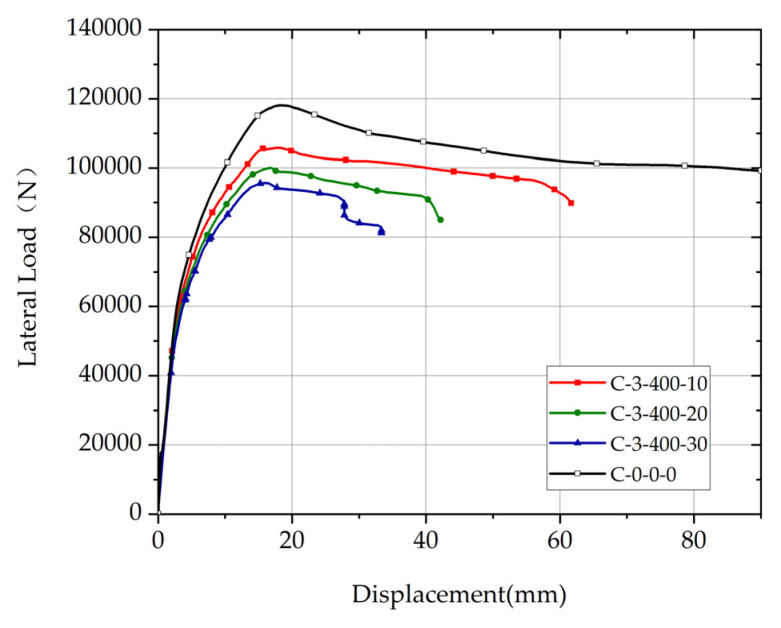
Comparison of load-displacement relationship curves of columns with different corrosion ratios.

Comparison of load-displacement relationship curves of columns with different rebar corrosion ratios of the corroded segment and the uncorroded column are shown in Figure 16. The position of the corroded segment is 3, the length of the corroded segment is 400 mm, and the rebar corrosion ratios of the corroded segment of columns are 10%, 20%, and 30%. The ultimate deformation of non-uniformly corroded columns decreases significantly with the increase of *η*.

**Figure 17:**
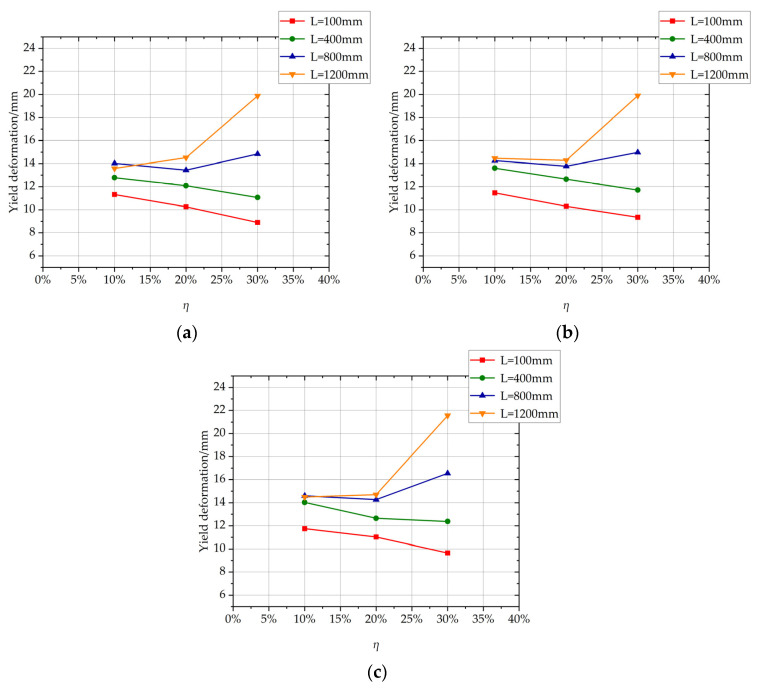
The influence of the rebar corrosion ratio of the non-uniformly corroded segments on the yield deformation of the columns with different *L* values. (**a**) *p* = 1; (**b**) *p* = 2; (**c**) *p* = 3.

The influence of the rebar corrosion ratio of non-uniformly corroded segments on the yield deformation of columns with different *L* values is shown in Figure 17. For columns with *L* of 100 mm or 400 mm, the yield deformation decreases approximately linearly with the increase of the rebar corrosion ratio of the corroded segment. For columns with *L* of 800 mm or 1200 mm, the yield deformation increases significantly with the increase of the rebar corrosion ratio of the corroded segment from 20% to 30%. It can be found that the reason for this phenomenon is that the significant bond-slip deformation between rebar and concrete occurs in the corroded segment with a long length at a high rebar corrosion ratio.

**Figure 18:**
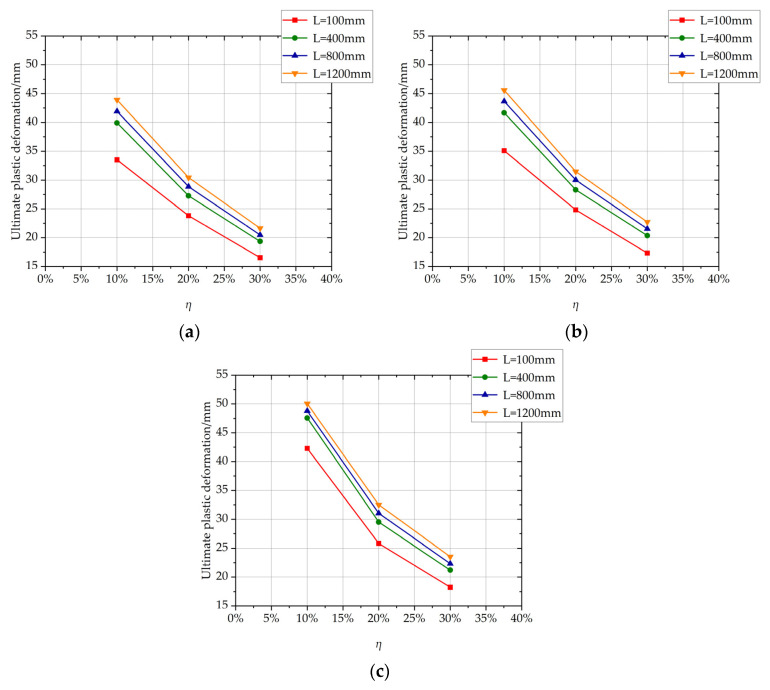
The influence of the rebar corrosion ratio of the non-uniformly corroded segments on the ultimate plastic deformation of columns with different *L* values. (**a**) *p* = 1; (**b**) *p* = 2; (**c**) *p* = 3.

The influence of the rebar corrosion ratio of the non-uniformly corroded segments on the ultimate plastic deformation of columns with different *L* values is shown in Figure 18. With the increase of the rebar corrosion ratio of the corroded segment, the ultimate plastic deformation decreases, and the descending trend of the ultimate plastic deformation slows down.

## 4. Conclusions

A numerical analysis of the deformation performance of longitudinal non-uniformly corroded RC columns was conducted with the aid of the ABAQUS software. Based on the numerical simulation results, the significant influence of corroded critical position on the deformation performance of longitudinal non-uniformly corroded RC columns was discovered, and the effects of position, length, and rebar corrosion ratio of the longitudinal non-uniformly corroded segment on the deformation performance of the corroded RC columns were analyzed. The following conclusions are drawn:

(1) The ultimate deformations of the longitudinal non-uniformly corroded columns with *p* exceeding *p_c_* are almost identical to that of the uncorroded column, and the ultimate deformations of the longitudinal non-uniformly corroded columns with *p* not exceeding *p_c_* are significantly smaller than that of the uncorroded column. The reason for the significant effect of the position of the corroded segment on the deformation performance of the non-uniformly corroded columns is that the yield condition of the rebar at the bottom section of the corroded segment determines the most unfavorable section of the column.

(2) The position, length, and rebar corrosion ratio of the corroded segments all have effects on the yield deformation of the non-uniformly corroded columns with *p* not exceeding *p_c_*, and the effect of length of the corroded segment on yield deformation is most significant, which is caused by the accumulation of the additional curvature and bond-slip deformation in the corroded segment along the lengthwise direction.

(3) With the increase of the position of the corroded segment from 1 to *p_c_* − 1, the ultimate plastic deformation increases approximately linearly. With the increase in the position of the corroded segment from *p_c_* − 1 to *p_c_*, the ultimate plastic deformation increases with a large increment, which is caused by extra plastic deformation at the bottom section of the column with *p* = *p_c_*.

(4) With the increase of the length of the corroded segment from 100 mm to 400 mm, the ultimate plastic deformation increases with a large increment, which is caused by plastic hinge development constrained by the length of the corroded segment. With the increase of the length of the corroded segment from 400 mm to 1200 mm, the ultimate plastic deformation increases slightly and approximately linearly.

(5) With the increase of the rebar corrosion ratio of the corroded segment, the ultimate plastic deformation decreases, and the descending trend of the ultimate plastic deformation slows down.

This study focuses on the effects of key corrosion parameters on the qualitative deformation performance of longitudinal non-uniformly corroded RC columns. The mathematical models for predicting the deformation performance of corroded RC columns quantitatively after considering the effects of all kinds of design parameters more comprehensively could be proposed further. This study did not consider the uncertainty of corrosion parameters on the deformation performance of the corroded RC column. It could be further investigated.

## Figures and Tables

**Table 1:** Comparison between test and simulation results.

Specimens	Test		Simulation		*E_F_*	*E*_∆_
	*F*_max_ (kN)	Δ_u_ (mm)	*F*_max_ (kN)	Δ_u_ (mm)		
S1	64.13	>90	64.75	>90	1.0%	-
S2	55.09	86.61	55.28	83.49	0.3%	−3.6%
S3	48.77	77.75	51.93	79.62	6.5%	2.4%
S4	46.68	37.50	47.07	39.74	0.8%	5.6%

^1^ *F*_max_ is the peak load of the specimen. ^2^ Δ_u_ is the ultimate deformation of the specimen, and the ultimate limit state is defined in Section 3.1. ^3^ *E_F_* is the error of the peak load of the simulation result on the peak load of the test result. ^4^ *E*_Δ_ is the error of the ultimate deformation of the simulation result on the ultimate deformation of the test result.

**Table 2:** The position, the length, and the rebar corrosion ratio of the corroded segment.

Parameters	Values
*p*	1, 2, 3, …, *p_max_*
*L*	100, 400, 800, 1200
*η*	10%, 20%, 30%

^1^ *p_max_* is the upper limit position of the corroded segment, which is determined by Equation (7).

## Data Availability

The original contributions presented in the study are included in the article, further inquiries can be directed to the corresponding author.
